# Genomic Comparison of Highly Virulent, Moderately Virulent, and Avirulent Strains From a Genetically Closely-Related MRSA ST239 Sub-lineage Provides Insights Into Pathogenesis

**DOI:** 10.3389/fmicb.2018.01531

**Published:** 2018-07-10

**Authors:** Jo-Ann M. McClure, Sahreena Lakhundi, Ayesha Kashif, John M. Conly, Kunyan Zhang

**Affiliations:** ^1^Centre for Antimicrobial Resistance, Alberta Health Services/Calgary Laboratory Services/University of Calgary, Calgary, AB, Canada; ^2^Department of Pathology and Laboratory Medicine, University of Calgary, Calgary, AB, Canada; ^3^Department of Microbiology, Immunology and Infectious Diseases, University of Calgary, Calgary, AB, Canada; ^4^Department of Medicine, University of Calgary, Calgary, AB, Canada; ^5^The Calvin, Phoebe and Joan Snyder Institute for Chronic Diseases, University of Calgary, Calgary, AB, Canada

**Keywords:** methicillin-resistant *Staphylococcus aureus* (MRSA), MRSA-ST239 lineage, pathogenesis, virulence, whole genome sequence (WGS), single nucleotide polymorphism (SNP), phylogenetic analysis, mobile genetic element (MGE)

## Abstract

The genomic comparison of virulent (TW20), moderately virulent (CMRSA6/CMRSA3), and avirulent (M92) strains from a genetically closely-related MRSA ST239 sub-lineage revealed striking similarities in their genomes and antibiotic resistance profiles, despite differences in virulence and pathogenicity. The main differences were in the *spa* gene (coding for staphylococcal protein A), *lpl* genes (coding for lipoprotein-like membrane proteins), *cta* genes (genes involved in heme synthesis), and the *dfrG* gene (coding for a trimethoprim-resistant dihydrofolate reductase), as well as variations in the presence or content of some prophages and plasmids, which could explain the virulence differences of these strains. TW20 was positive for all genetic traits tested, compared to CMRSA6, CMRSA3, and M92. The major components differing among these strains included *spa* and *lpl* with TW20 carrying both whereas CMRSA6/CMRSA3 carry *spa* identical to TW20 but have a disrupted *lpl*. M92 is devoid of both these traits. Considering the role played by these components in innate immunity and virulence, it is predicted that since TW20 has both the components intact and functional, these traits contribute to its pathogenesis. However, CMRSA6/CMRSA3 are missing one of these components, hence their intermediately virulent nature. On the contrary, M92 is completely devoid of both the *spa* and *lpl genes* and is avirulent. Mobile genetic elements play a potential role in virulence. TW20 carries three prophages (ϕSa6, ϕSa3, and ϕSPβ-like), a pathogenicity island and two plasmids. CMRSA6, CMRSA3, and M92 contain variations in one or more of these components. The virulence associated genes in these components include staphylokinase, entertoxins, antibiotic/antiseptic/heavy metal resistance and bacterial persistence. Additionally, there are many hypothetical proteins (present with variations among strains) with unknown function in these mobile elements which could be making an important contribution in the virulence of these strains. The above mentioned repertoire of virulence components in TW20 likely contributes to its increased virulence, while the absence and/or modification of one or more of these components in CMRSA6/CMRSA3 and M92 likely affects the virulence of the strains.

## Introduction

Methicillin-resistant *Staphylococcus aureus* (MRSA) continues to be a major cause of hospital infection, as well as an emerging cause of community associated infections (David and Daum, 2010; Kock et al., 2010). Enright et al. (2002) revealed that the majority of global MRSA clones belonged to one of the five major clonal complexes (CCs) including CC5, CC8 (including CC8-ST239 sub-group), CC22, CC30 and CC45, however, strains belonging to many other CCs are emerging as significant sources of infection. Within these CCs, ST239 carrying staphylococcal cassette chromosome *mec* (SCC*mec*) III, is a healthcare-associated MRSA lineage present worldwide (Aires de Sousa and de Lencastre, 2004; Harris et al., 2010; Gray et al., 2011; Hsu et al., 2015). ST239-MRSA-III is prevalent in Asia, South America and Eastern Europe, and includes strains like the Brazilian, Hungarian, Portuguese, AUS-EMRSA-2 and 3, Viennese and EMRSA-1, -4,-7, 9, and 11 clones (Aires de Sousa and de Lencastre, 2004; Conceicao et al., 2007; Monecke et al., 2011). Phylogenetic analysis has revealed the intercontinental dissemination and hospital transmission of CC8-ST239 isolates throughout North America, Europe, South America, and Asia (Harris et al., 2010; Wang et al., 2012). In the 1990s, ST239 dispersed from South America to Europe and from Thailand to China (Gray et al., 2011).

Holden et al. (2010) isolated a highly transmissible outbreak MRSA strain, TW20, belonging to ST239-MRSA-III from an intensive care unit (ICU) in a London hospital. TW20 was found to be a highly invasive MRSA strain, with its acquisition four times more likely to result in bacteremia as compared to the other epidemic strains like EMRSA-15 or -16. In addition, it was more frequently isolated from vascular device cultures, had an extended antibiotic resistance pattern and an elevated minimum bactericidal concentration for chlorhexidine compared to other MRSA strains. TW20 was consequently classified as a highly virulent MRSA strain. Interestingly, the association of this strain with carriage/colonization sites like the nares, axilla and perineum was less frequent, suggesting a difference in its colonization capability as compared to other MRSA strains (Holden et al., 2010). The authors investigated the genetic basis for this increased transmissibility, resistance and virulence by analyzing its whole genome and comparing it with other MRSA lineages. They identified two large mobile genetic regions, a wide range of genes responsible for antibiotic, antiseptic and heavy metal resistance, as well as mutations in some housekeeping genes, which could all be responsible for its increased virulence, invasiveness and survival in the hospital environment (Holden et al., 2010).

**FIGURE 1:**
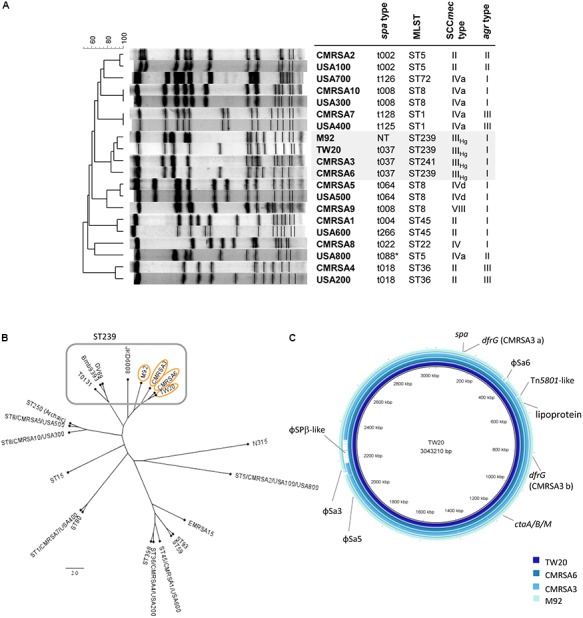
The ST239 strains are genetically closely-related and form a ST239 sub-lineage distinct from other national and international MRSA lineages. **(A)** Pulsotype and molecular characterization of the ST239 isolates and their relatedness to other Canadian (CMRSA1-10) and United States (USA1000-800) epidemic strains; *spa*, staphylococcal protein A; MLST, multilocus sequence typing; SCC*mec*, Staphylococcal cassette chromosome *mec*; *agr*, accessory gene regulator; NT, non-typeable. **(B)** Single nucleotide polymorphism (SNP)-whole genome sequence (WGS) Phylogenetic cladogram showing relatedness of the ST239 isolates to other international ST239 strains, as well as some of the major MRSA lineages distributed worldwide. ST239 isolates are clustered together with TW20, CMRSA6, CMRSA3, and M92 forming a ST239 sub-lineage. Strains (common name; GenBank accession numbers) are as follows: TW20 (FN433596), CMRSA6 (CP027788), CMRSA3 (CP0209685), M92 (CP015447), JKD6008 (CP002120), GV69 (CP009681), Bmb9393 (CP005288), T0131 (CP002643), CMRSA5/USA500 (2395; CP007539), ST250 (Archaic, COL; CP000046), CMRSA10/USA300 (FPR3757; CP000255), ST15 (08-02119; CP015645), CMRSA7/USA400 (MW2; BA000033), ST80 (11819-97; CP003194), ST398 (S0385; NC017333), CMRSA4/USA200 (MRSA252; BX571856), CMRSA1/USA600 (CA-347; CP006044), ST59 (SA40; CP003604), ST93 (JKD6159; CP002114), EMRSA-15 (H-EMRSA-15; CP007659), CMRSA2/USA100/USA800 (Mu50; BA000017), N315 (BA000018). **(C)** Blast ring image generator (BRIG) analysis of the ST239 genomes showing unconserved regions between the genomes, as indicated by labels. Rings from inner to outer (and darkest blue to lightest blue) are as follows: TW20, CMRSA6, CMRSA3, M92.

In Canada, we have had several epidemic hospital-associated MRSA (HA-MRSA) strains (**Figure 1A**) identified. These include CMRSA6 and CMRSA3 (ST239-t037-MRSA-III and ST241-t037-MRSA-III respectively which are similar to USA epidemic pulsotype strain USA700) which before the emergence of community-associated MRSA (CA-MRSA), along with CMRSA2 (ST5-t002-MRSA-II, similar to USA100/800), were the dominant causes of healthcare-associated MRSA infections in Canada. CMRSA2 was the predominantly isolated HA-MRSA strain during 2000–2006, while CMRSA6 (replacing CMRSA3) was the most common strain isolated in the hospital in later years (Christianson et al., 2007). Compared to CMRSA2, infections caused by CMRSA6 and CMRSA3 were less frequent and less severe and/or invasive (Christianson et al., 2007). In a *Caenorhabditis elegans* MRSA virulence infection model, CMRSA6 and CMRSA3 killed only a small percentage of worms compared to CMRSA2, thereby classifying them as non- or low-nematocidal MRSA strains, which correlated well with clinically invasive anatomic site data from another study (Wu et al., 2010). CMRSA6 and CMRSA3 are therefore considered as moderately virulent strains.

In the late 1980s, M92, a colonizing MRSA strain and close relative of CMRSA6 and CMRSA3, was isolated from a hospital site in Calgary, AB, Canada. Over the course of many years, M92 was frequently found to be associated with nasal colonization in hospital staff and patients but was never found associated with infections (Wu et al., 2010; McClure and Zhang, 2017). In the *C. elegans* model, this benign strain did not show any nematocidal activity and was, therefore, classed as an avirulent strain and used as a control in many infection models (Wu et al., 2010, 2012a,b).

A whole genome comparison of TW20, CMRSA6, CMRSA3, and M92 revealed striking similarities. Sequence alignments of the genomes indicated that they are very closely related with only minor differences, which contrasts significantly to the virulence of these strains in clinical scenarios, with TW20 being highly virulent, CMRSA6 and CMRSA3, moderately virulent and M92, avirulent. While the differences are few, they likely represent genetic components which impact pathogenesis and could explain the virulence differences observed among the strains, both *in vivo* and in the clinical setting. Here we present whole genome sequence (WGS) comparisons among TW20, CMRSA6, CMRSA3 and M92, highlighting the factors that could play a role in the virulence and pathogenicity differences noted in these strikingly similar strains.

## Materials and Methods

### Bacterial Strains

The Canadian epidemic MRSA reference strains CMRSA1 to 10 (including CMRSA6 and CMRSA3) were provided by the National Microbiology Laboratory, Health Canada, Winnipeg, MB, Canada. The United States epidemic MRSA reference strains USA100 to USA800 (NRS382, NRS383, NRS384, NRS123, NRS385, NRS22, NRS386, and NRS387, respectively) were obtained through the Network on Antimicrobial Resistance in *Staphylococcus aureus* Program (NARSA) supported under NIAID/NIH contract no. N01-AI-95359. Strain M92 was kindly provided by Dr. T. Louie at University of Calgary, Canada, and strain TW20 by Dr. Julian Parkhill at the The Wellcome Trust Sanger Institute, United Kingdom.

### Strain Molecular and Phenotypic Characterization

Staphylococcal isolates were fingerprinted by pulsed field gel electrophoresis (PFGE) after digestion with *Sma*I following a standardized protocol (Mulvey et al., 2001). PFGE-generated DNA fingerprints were digitized and analyzed with BioNumerics Ver. 6.6 (Applied Maths, Sint-Martens-Latem, Belgium) by using a position tolerance of 1.0 and an optimization of 1.0. Isolates were further characterized with Staphylococcal protein A (*spa*) typing (Harmsen et al., 2003), multilocus sequence typing (MLST) (Enright et al., 2000), SCC*mec* typing (McClure et al., 2010; Zhang et al., 2012), and accessory gene regulator (*agr*) typing (Peacock et al., 2002). Screening for antibiotic-resistant phenotypes was performed by use of VITEK 1 (bioMerieux) and the Clinical and Laboratory Standards Institute oxacillin agar screen, while confirmation of methicillin resistance was achieved using an in-house polymerase chain reaction (PCR) assay for the *mecA* gene (McClure et al., 2010; Zhang et al., 2012). Antibiotic susceptibility data for strain TW20 was obtained from the Holden study (Holden et al., 2010).

### DNA Sequencing and Whole Genome Sequence Analysis

Genomic DNA for strains CMRSA6, CMRSA3, and M92 was isolated by phenol:chloroform extraction and sequenced with Pacific Biosciences (PacBio) RSII sequencing technology (McGill University Génome Québec Innovation Centre), as well as with Illumina MiSeq technology (Core DNA sequencing services, University of Calgary). Hybrid sequence assembly was performed using both read sets and the genomes annotated with NCBI’s Prokaryotic Genomes Annotation Pipeline and deposited under accession numbers CP027788, CP029685, and CP015447, respectively. The genomes of TW20 were available under accession numbers FN433596 and CP015447, and the *spa* gene sequence of *S. aureus* strain 8325-4 was available under accession number J01786. Single nucleotide polymorphism (SNP) WGS phylogenetic analysis was performed using CSI Phylogeny v1.4 with default settings, using strain N315 (BA000018) as the reference and rooting genome (Center for Genomic Epidemiology, Kongens Lyngby, Denmark). Phylogenetic trees were visualized with FigTree v1.4.3 (Institute of Evolutionary Biology, University of Edinburgh, Edinburgh, United Kingdom). Blast ring images were generated using BRIG v0.95 (Alikhan et al., 2011). Representative genomic structures were generated and analyzed with Vector NTI Advance v11.5.2 (Invitrogen), DNA multiple sequence alignment with TCoffee (Notredame et al., 2000; Wallace et al., 2006; Moretti et al., 2007; Di Tommaso et al., 2011), and protein translation using fr33.net (France). Prophage identification and annotation was conducted using PHASTER software (Zhou et al., 2011; Arndt et al., 2016), and the comparisons using Easyfig (Sullivan et al., 2011).

## Results and Discussion

### TW20, CMRSA6, CMRSA3, and M92 Form a Genetically Closely-Related ST239 Sub-lineage

Pulsed field gel electrophoresis analysis of TW20 revealed that it clustered together with CMRSA6, CMRSA3, and M92 when compared against major United States/Canada epidemic strains (**Figure 1A**). Molecular characterization of TW20, CMRSA6, CMRSA3, and M92 showed that they all carry SCC*mec* type III_Hg_ and *agr* type I (**Figure 1A**), as well as the same *spa* type (t037), with the exception of M92 which is non-typeable via *spa* typing (**Figure 1A**). The strains belong to ST239, a major dominant hospital associated MLST type, except for CMRSA3 which belongs to the closely related ST241. ST241 differs from ST239 by a single point mutation at 268 bp in the *yqiL* locus (coding for acetyl coenzyme A acetyltransferase) whereby adenine in ST239 is replaced by a guanine (A to G). CMRSA3 is consequently very closely related to TW20, CMRSA6, and M92 (**Figure 1A**).

The genome of TW20 was published and is available (Holden et al., 2010), therefore whole genome sequencing was done on CMRSA6, CMRSA3, and M92 in order to analyze and compare the genomes of all four strains of this ST239 sub-lineage. The genome of TW20 is 3.0 Mbp and is reported to be the largest sequenced *S. aureus* genome. The genome of CMRSA6, CMRSA3, and M92 are similar in size, being 3.0, 2.9, and 3.0 Mbp, respectively. SNP whole genome phylogenetic analysis of TW20, CMRSA6, CMRSA3, and M92 supported the close genetic relatedness of these strains. They clustered apart from the other dominant global MRSA lineages (such as ST59 EMRSA-15, and USA300) and clustered together with international ST239 isolates (like GV69, T0131, and JKD6008) but forming a distinct sub-lineage within the ST239 (**Figure 1B**).

Detailed analysis of the TW20 genome was performed by Holden et al. (2010), and they suggested that several components in the genome could play a role in its highly virulent nature. These components included two large regions of 635 and 127 kb, as well as genes coding for QacA (antiseptic resistance protein), CadA (cadmium-transporting ATPase), TetM (tetracycline resistance protein), and DfrG (trimethoprim-resistant dihydrofolate reductase). Genes carried on mobile genetic elements like prophages (such as SCIN, *sek* and *sea*) or a pathogenicity island (such as *entK*, *entQ*) were also suspected as being responsible for the high virulence. A surface anchored protein with the LPxTG motif, as well as point mutations in housekeeping genes coding for DNA gyrase subunit A (Ser84Leu; shown to confer resistance to quinolones) and isoleucyl-tRNA synthetase (Val588Phe; shown to confer low level mupirocin resistance), were also mentioned.

Comparisons were made between the genomes of the highly virulent TW20, the moderately virulent CMRSA6 and CMRSA3, and the avirulent M92, with respect to all regions mentioned by Holden et al. (2010). Careful analysis revealed that the above mentioned elements were, for the most part, present and similar among the four strains and, therefore, less likely to account for the increased virulence of TW20. Our analysis of the four genomes did reveal regions that differed between the ST239 strains, and these regions could be responsible for the elevated virulence of TW20. Differences were noted in *spa* (coding for staphylococcal protein A, SpA, a wall anchored protein imparting *S. aureus* with the ability to avoid opsonins present in normal serum), the *lpl* gene (coding for lipoprotein-like membrane proteins with N-terminal lipid moiety anchoring it to the outer leaflet of the cytoplasmic membrane), the *cta* genes (genes involved in heme synthesis), which were not identified as the virulent factors in Holden’s study (Holden et al., 2010), as well as, *dfrG*/conserved hypothetical protein genes (coding for trimethoprim-resistant form of dihydrofolate reductase), which was pointed out by Holden et al. (2010) (**Figure 1C**). In addition, there were variations in the presence or content of prophages, including ϕSa6/5 (ϕSa6 formerly classified as ϕSa1 in TW20), ϕSa3 and ϕSPβ-like (**Figure 1C**). Furthermore, CMRSA3 and TW20 carried plasmids which contained similar content and were found to be missing in CMRSA6 and M92. Each of these unique regions within the chromosome was studied in detail to understand its possible role in the virulence and pathogenicity in *S. aureus*.

### *spa* Gene Truncation and SpA Functional Destitution in M92

**FIGURE 2:**
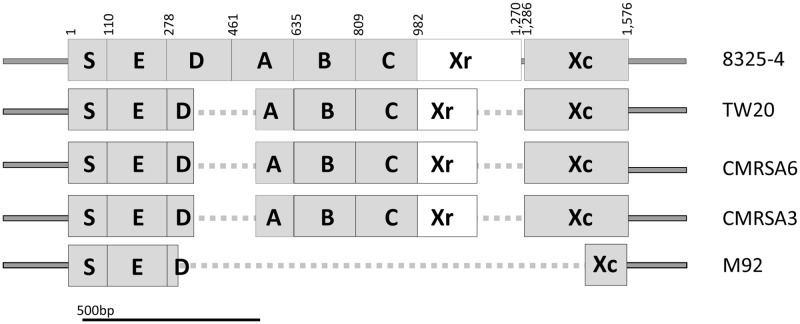
Structural and sequence comparison of the *spa* genes among the ST239 isolates shows deletions in TW20, CMRSA6, CMRSA3, and M92. Structural arrangement of the complete *spa* gene in strain 8325-4 (Accession number J01786) showing regions coding for the signal peptidase domain (S), IgG binding domains (E, D, A, B, C), the short sequence repeat region (Xr), and the anchoring domain (Xc). Deletions in the *spa* gene of TW20, CMRSA6, CMRSA3, and M92 are mapped and indicated with a dotted line. The Xr regions differ between the strains and is the basis for their assignment to different *spa* types. Nucleotide positions in 8325-4 are indicated at the top.

SpA, encoded by *spa*, plays an important role in the virulence and pathogenicity of *S. aureus* through innate immune evasion. The prototype *spa* gene from *S. aureus* strain 8325-4 contains an open reading frame (ORF) consisting of 1576 bp, giving rise to a protein of M_r_ = 58,703 (Uhlen et al., 1984), and is considered to be a complete *spa* gene. As seen in **Figure 2**, SpA of strain 8325-4 is composed of multiple domains, including the signal peptide (S) at the N terminus, responsible for directing the protein to its destination, followed by five highly homologous repeated domains (E, D, A, B, and C), which correspond to the immunoglobulin (Ig) binding domains and are found to vary in different *S. aureus* strains. The Xr region (also called the cell wall binding domain) follows, consisting of variable numbers of repeats, approximately eight amino acids in length, which form the basis of *spa* typing. Finally there is the X_C_ region, which is a transmembrane domain consisting of a LPxTG motif, a hydrophobic region and a charged tail (Schneewind et al., 1993). It plays an important role in anchoring the protein to the surface of the cell (Schneewind et al., 1992, 1993).

The *spa* genes of TW20 (SATW20_01230), CMRSA6 and CMRSA3 are identical to each other at 1,283 bp in length. They contain complete E, B, C and Xc domains, identical to those of 8325-4 (**Figure 2**). However, in all three strains the D and A domains are partially missing (from 342 to 515 bp) (**Figure 2**). TW20, CMRSA6 and CMRSA3 also differ from 8325-4 in their Xr regions, both in terms of sequence composition and length (184 bp as compared to the 289 bp of strain 8325-4), which forms the basis for their differential *spa* classifications. In strain M92, by contrast, the majority of the *spa* gene is truncated, leaving a gene of 425 bp in length. Domains S and E are present, while domains A, B, and C are completely missing and only 25 bp of domain D is present, likely making it non-functional (**Figure 2**). While the E domain is present in M92 *spa* gene, its role as an Ig binding domain is controversial. Earlier studies have indicated that domain E has diverged more than the other four domains (A–D) and therefore probably has a different biological function instead of Ig binding (Hjelm et al., 1975; Sjodahl, 1977a,b; Wright et al., 1977; Hanson and Schumaker, 1984; Uhlen et al., 1984). Domain Xr is completely missing in M92 making it non-typeable via *spa* typing. The majority of the N-terminal part (168 bp out of 291 bp) of domain Xc is missing as well (**Figure 2**). This means that the LPxTG motif, hydrophobic domain and probably a part of charged tail are missing from M92 *spa* and the protein may not anchor to the surface of the cell. Thus, M92 *spa* may potentially be devoid of any function of SpA.

SpA can be regarded as an innate immune evasion molecule, conferring the ability to survive within the host and cause successful infection, and has been shown to be present in 98% of coagulase positive *S. aureus* strains (Forsgren, 1970). The immunoglobulin binding domain of SpA has the affinity to bind to both the Fc portion of IgG, as well as the Fab portion of the V_H_3 region of IgM located on the surface of B cells. Because SpA normally resides on the organism’s cell surface, the interaction of IgG with SpA results in coating of the pathogen’s cell surface with IgG molecules. These IgG molecules are in the incorrect orientation for recognition by the Fc receptors of neutrophils, inhibiting opsonophagocytic killing of the organism (Foster, 2005; Rooijakkers et al., 2005). On the other hand, the ability of SpA to bind to the Fab portion of the V_H_3 region of IgM on the surface of B cells causes the cells to proliferate and undergo apoptosis. This diminishes the repertoire of antibody-secreting B lymphocytes in the spleen and bone marrow (Goodyear and Silverman, 2004). As mentioned, the SpA of TW20, CMRSA6, and CMRSA3 contain complete immunoglobulin binding domains B, C, and potentially E. Although domains A and D are partial and likely non-functional, these strains still have 2 or 3 complete Fc and Fab binding regions, playing a role in protecting these strains from the host immune system. In contrast, the SpA of M92 is completely missing domains A, B and C, with only 25 bp of domain D present and is likely non-functional. While domain E is present, its controversial role as an Ig binding domain possibly leaves M92 *spa* devoid of any IgG binding domains. In addition, due to the lack of a LPxTG motif, SpA from M92 is probably not expressed on the cell surface. As a consequence, once inside the human body, a lack of functional SpA on M92’s cell surface might result in its opsonization and killing by human immune cells.

### *lpl* Disruption in CMRSA6, CMRSA3 and M92, but Not in TW20

TW20, CMRSA6, CMRSA3 and M92, all belong to the clonal complex CC8, most of which carry a conserved genomic island, *ν*Saα (Babu et al., 2006). This genomic island is characterized by two clusters of tandem repeat sequences, including an exotoxin (*set*) and a number of homologous lipoproteins (Lpp) arranged in tandem and referred to as lipoprotein-like (*lpl*) (Babu et al., 2006; Baba et al., 2008; Tsuru and Kobayashi, 2008). The exact function of these lipoproteins (Lpl)is not known, however they have recently been shown to trigger host cell invasion, increase pathogenicity and may contribute to the epidemic nature of CC8 and CC5 strains (Nguyen et al., 2015).

**FIGURE 3:**
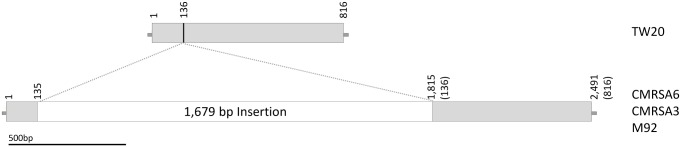
The lipoprotein sequence of TW20 is compared to the tandem lipoproteins in CMRSA6, CMRSA3, and M92. Structure of the TW20 lipoprotein gene as compared to the tandem lipoprotein genes in CMRSA6, CMRSA3 and M92, which are identical and contain a 1,679 bp insertion. The location of insertion relative to the TW20 sequence is marked with a line and falls between base pairs 135 and 136. Numbers indicate nucleotide locations within the DNA sequence, while numbers in brackets for CMRSA6, CMRSA3, and M92 represent corresponding nucleotides in the TW20 gene.

The *lpl* of TW20 (SATW20_05130) is a 816 bp sequence which results in the transcription of a protein with 271 amino acids (**Figure 3**). The transcribed protein from TW20 resembles Lpl and proteins containing conserved motifs called DUF567 (domain of unknown function). The *lpl* of CMRSA6, CMRSA3 and M92, in contrast, have 1,679 bp of DNA inserted within *lpl* (**Figure 3**), resulting in a gene size of 2,491 bp. The insertion is at bp 136 in the TW20 gene (as indicated by a line in **Figure 3**) and is possibly due to homologous recombination. The first 136 bp of sequence in TW20, CMRSA6, CMRSA3 and M92 are all identical, as are the next 36 bps (sequence: GAACAAATCAAAAAGAGCTTTGCGAAAACATTAGAT) of both the normal TW20 *lpl* and the inserted DNA. This 36 bp sequence may represent a region where homologous recombination occurred, permitting incorporation of the extra DNA into the *lpl* genes of CMRSA6, CMRSA3, and M92. Regardless of the mechanism involved, incorporation of the extra DNA results in disruption of the original *lpl* gene. Fortunately, the interruption continues in the same ORF as the original gene and transcription of the gene in CMRSA6, CMRSA3, and M92 results in a new Lpl. This new Lpl is 271 amino acids long, but is missing a large part of the C-terminus of the original *lpl*, which may have functioned as a cell wall anchor. Interestingly, following the new Lpl lies a second ORF of 488 bp, which could code for a protein of 161 amino acids. However, this frame does not contain a start codon and is therefore unlikely to be transcribed into a protein.

Of interest is the fact that the genomes of some virulent strains, like Newman, can contain both types of *lpl* sequences; the original one similar to TW20, as well as the one with the insertion similar to CMRSA6, CMRSA3, and M92. We did not, however, find the inserted (1,679 bp) sequence anywhere within the TW20 genome, nor was the original *lpl* without insertion found in the genomes of the other three strains. It is also interesting to note that Lpl and the proteins containing DUF567 have been found to be taxonomically restricted to staphylococci and have recently been shown to play a significant role in the pathogenicity and virulence of *S. aureus* USA300 (Nguyen et al., 2015; Shahmirzadi et al., 2016).

Nguyen et al. (2015) deleted the entire *lpl* gene cluster in *S. aureus* USA300, making the mutant strain less invasive, with decreased ability to stimulate pro-inflammatory cytokines compared to the original or complemented strain. Invasiveness helps a pathogen shield itself from the harmful effect of antimicrobials, as well as from the human immune system, thereby contributing to its virulence and pathogenesis. TW20 with its intact Lpl may have an increased ability to stimulate the production of pro-inflammatory cytokines and may have increased invasiveness, as compared to CMRSA6, CMRSA3 and M92 with their interrupted *lpl* genes.

### Potential Disruption of *Cta* in CMRSA6

**FIGURE 4:**
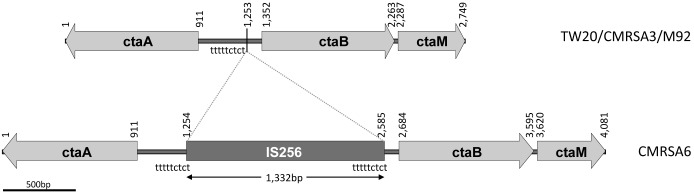
*ctaA*, *ctaB*, and *ctaM* sequence comparison among the ST239 isolates showing an IS256 insertion in CMRSA6. The structural arrangement of the *ctaA/B/M* genes is identical in strains TW20, CMRSA3 and M92, while CMRSA6 shows a 1,332 bp insertion of the IS256 transposase between the *ctaA* and *ctaB* genes. Nucleotide positions of the region are noted above, with the location of the insertion marked by a line at position 1,253 bp. The locations of the repeat sequence “tttttctct” are indicated.

In gram positive bacteria, heme synthesis is an important pathway providing substrate for the production of terminal oxidases (Mogi et al., 1994). In a heme-iron deficient environment, *S. aureus* fulfills its iron requirement via a complex pathway involving several genes (*cta*) coding for enzymes required for the synthesis of heme A (Hammer et al., 2016). The *cta* genes include *ctaA* (911 bp, coding for CtaA of 302 amino acids), *ctaB* (912 bp, coding for CtaB of 303 amino acids), and *ctaM* (463 bp, coding for CtaM of 153 amnio acids) (**Figure 4**). The orientation of *ctaA* and *ctaB* is in opposite direction to each other, with 441 bp between them, while *ctaM* is oriented in the same direction as *ctaB* (**Figure 4**). Whole genome analysis of TW20, CMRSA3, and M92 revealed that the *cta* genes in these strains are identical. The *cta* genes of CMRSA6, in contrast, have an IS256 insertion of 1,332 bp within the 441 bp region between *ctaA* and *ctaB*, with characteristic repeat regions (TTTTCTCT) at 1,253 bp (**Figure 4**). It is noteworthy that this insertion did not disrupt any gene, however, we are not sure if it resulted in the disruption of promotor function for any *cta* genes, as no promotor information is available. This promotor or promotors, if present, may be controlling the transcription of a single, or multiple *cta* genes, meaning that disruption in promotor function could potentially lead to the loss of the expression of one or multiple *cta* genes. All these genes play an important role in heme synthesis; Heme B is converted to heme O via CtaB which is then converted to heme A via CtaA (Svensson et al., 1993; Svensson and Hederstedt, 1994; Clements et al., 1999). CtaM was recently shown to support the function of QoxABCD, a respiratory oxygen reductase (Hammer et al., 2016). Therefore, the loss of even a single gene expression might affect the function of other genes.

Several studies have demonstrated that mutations in the *cta* genes results in decreased ability of the organism to survive long term starvation (Clements et al., 1999), decreased pigment production, attenuation of hemolytic activity and decreased growth (Lan et al., 2010; Xu et al., 2016). It also resulted in decreased transcription of several virulence genes, thereby affecting virulence (Xu et al., 2016), as well as host specific organ colonization of the organism (Hammer et al., 2013, 2016).

### Presence of DfrG, Conferring Trimethoprim Resistance, in TW20, CMRSA6, and CMRSA3

**FIGURE 5:**
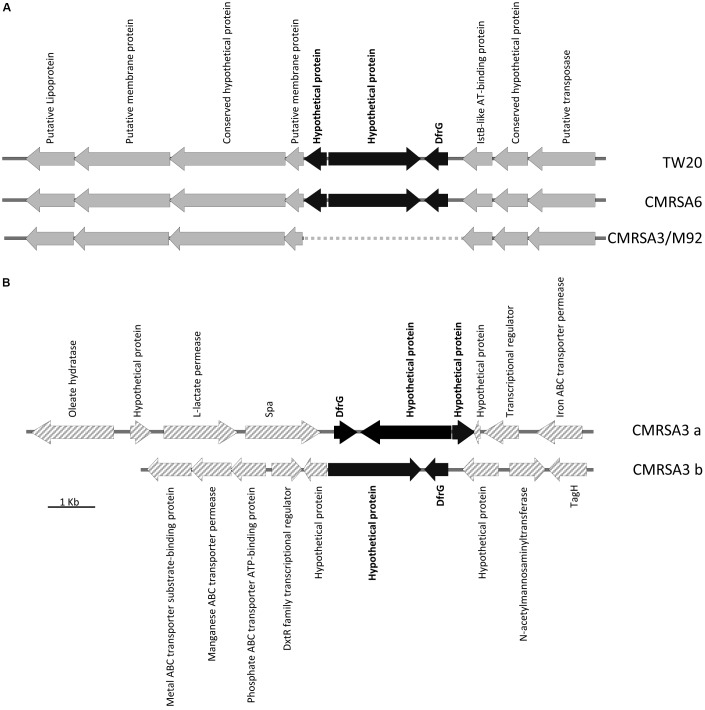
Insertion of the trimethoprim resistance gene, *dfrG*, in ST239 strains. **(A)** Partial Tn*5801*-like element structure demonstrating insertion of the *dfrG* gene, plus 2 genes coding for hypothetical proteins (colored black), in strains TW20 and CMRSA6. The insertion is absent in CMRSA3 and M92. Open reading frames (ORFs) surrounding the insertion site are indicated in gray. **(B)** CMRSA3 contains two copies of the *dfrG* and hypothetical protein genes, but located in two separate locations on the chromosome. ORFs surrounding the insertions are indicated by gray hatch. Protein products of each gene are listed.

DfrG, a trimethoprim-resistant dihydrofolate reductase, confers resistance to the antibiotic trimethoprim, used for the treatment of *S. aureus* infections (Rouch et al., 1989). The genome of TW20 and CMRSA6 carry a 31.3 kb region of Tn*5801*-like element (**Figure 5A**), which is similar to transposons ICEs (integrative and conjugative elements) found in the genome of other *S. aureus* strains like Mu50 (Kuroda et al., 2001) and Mu3 (Neoh et al., 2008). The Tn*5801*-like element is responsible for dissemination of the tetracycline resistance gene, *tetM*, which codes for a ribosomal protection protein conferring resistance to the action of tetracycline (de Vries et al., 2016). Within this Tn*5801*-like element in TW20 and CMRSA6, three additional genes have been observed, including *dfrG* (SATW20_04710), coding for trimethoprim-resistant dihydrofolate reductase, as well as two others coding for hypothetical proteins (**Figure 5A**). *dfrG* is 498 bp in length, coding for a protein of 165 amino acids long, while the other two genes are 1953 and 481 bp long, coding for a protein sequences of 650 and 159 amino acids in length (**Figure 5A**).

The genomes of CMRSA3 and M92 also carry the Tn*5801*-like element, however, the element in these strains is missing the three additional genes (*dfrG* and two hypothetical proteins) (**Figure 5A**). Interestingly, the genome of CMRSA3 still carries the genes for DfrG and both hypothetical proteins, but not associated with the Tn*5801* like element (**Figure 5B**). In fact, CMRSA3 carries two copies of the *dfrG* gene located at two different positions within its genome (**Figure 1C**). One copy is located adjacent to the 3′ end of the *spa* gene and is accompanied by both of the hypothetical proteins (CMRSA3a), while the other copy is found at approximately 810 kb and only accompanied by the larger (1953 bp) hypothetical protein (CMRSA3b) (**Figure 5B**). The copy of *dfrG*/hypothetical protein present at 810 kb is oriented in the same direction as the one in TW20 and CMRSA6, but the copy of *dfrG*/hypothetical proteins positioned near *spa* is oriented in the opposite direction to that in TW20 and CMRSA6 (**Figure 5B**).

Trimethoprim is an important antibiotic used for the treatment of staphylococcal infections, particularly skin and soft tissue infections (Rouch et al., 1989; Nathwani et al., 2008; Stevens et al., 2014). It acts by inhibiting an enzyme (dihydrofolate reductase) involved in the folate synthesis pathway (Miovic and Pizer, 1971). TW20, CMRSA6, and CMRSA3 all carry this trimethoprim-resistant dihydrofolate reductase enzyme and were phenotypically resistant to trimethoprim (**Table 1**), which is likely one of the factors contributing to survival and persistence of these strains in the high antibiotic selective pressures seen in hospitals. M92, devoid of this enzyme, is susceptible to the action of trimethoprim, which is confirmed in the antibiotic resistance profiles of the strains (**Table 1**). The function and significance of the hypothetical proteins present next to *dfrG* are unknown.

**Table 1:** Antibiotic resistance profiles.

	Ampicillin	Amp/sul	β-lactam	Cefazolin	Penicillin	Oxacillin	Gentamicin	Erythromycin	Tetracycline	Ciprofloxacin	Clindamycin	Trimethoprim	TMP-SMX	Rifampin	Vancomycin	Nitrofurantoin	Neomycin
CMRSA6	R	R	P	R	R	R	R	R	R	R	R		R	S	S	S	
CMRSA3	R	R	P	R	R	R	S	R	R	R	R		R	S	S	S	
M92	R	R	P	R	R	R	R	R	R	R	R		S	S	S	S	
TW20					R	R	R	R	R	R		R					R

*R, resistant; P, positive; S, sensitive; Amp/sul, ampicillin/sulbactam; TMX-SMX, trimethoprim/sulfamethoxazole.*

### Prophages and Mobile Genetic Elements Present in the ST239 Isolates

**FIGURE 6:**
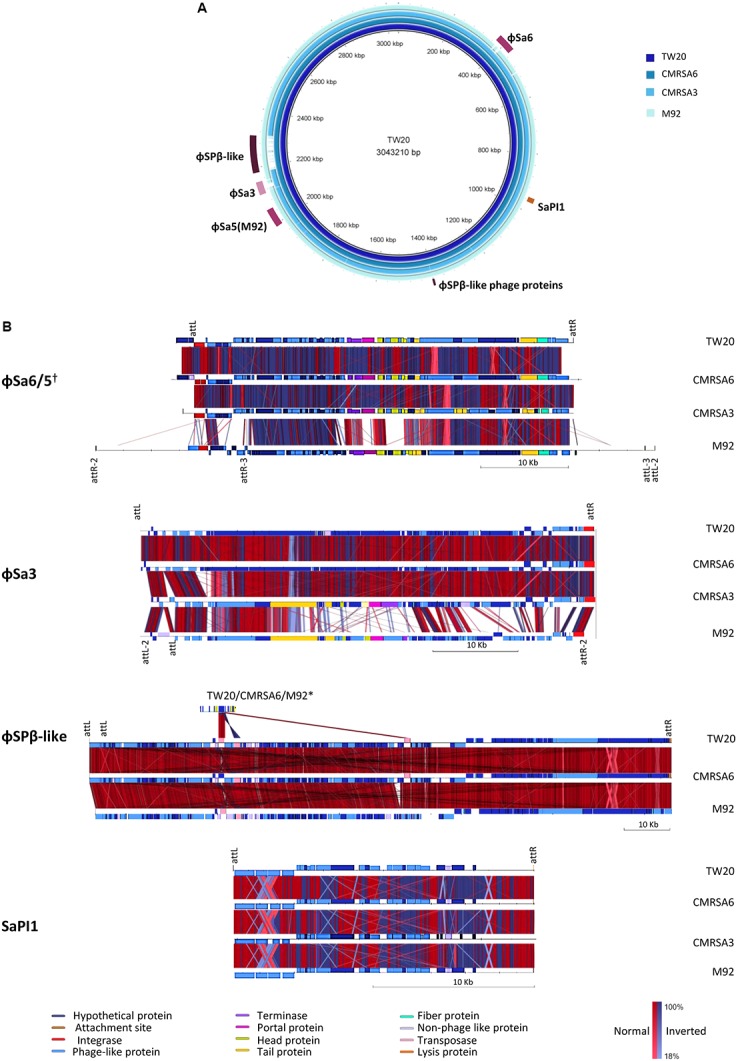
Six regions containing phages or mobile genetic elements are found in the ST239 isolates, with variations in their content. **(A)** BRIG alignment of the ST239 strains, with the locations of each MGE indicated. Rings from inner to outer (and darkest blue to lightest blue) are as follows: TW20, CMRSA6, CMRSA3, M92. **(B)** Comparisons of each mobile element region, with components differentiated by color. Homologies are marked with red for normal and blue for inverted, with color saturation indicative of percentage homology. Left and right MGE attachment site locations are indicated above the alignments, with markings below a strain representing differences within that strain. Attachment site sequences are as follows: ϕSa6/5 attL/R-AAAAAAGGGCAGA, attR2/L2-CTTTTTAAAATTA, attR3/L3-TAATTTAGTTAT; ϕSa3 attL/R-AAGTTGCAACAC, attL2/R2-AAAAATAATTAG; ϕSPβ-like attL/R- ATTATTATAATT; SaPI1 attL/R- TTGAAAATAAAA. Strains not depicted are missing the MGE in question. †While ϕSa6 of TW20, CMRSA6, and CMRSA3 are found in different genomic locations than ϕSa5 of M92, the phages are similar and included in the same comparison. ^∗^The ϕSPβ-like phage proteins in TW20, CMRSA6, and M92 are compared to ϕSPβ-like.

The staphylococcal genome displays several large sequence blocks with high variability which can carry determinants for antibiotic resistance and/or virulence. These variable regions can be classified as prophages, pathogenicity islands or staphylococcal cassette chromosomes (Baba et al., 2008), and several of them are present in the genomes of all four ST239 isolates studied here. Using the online phage search tool, PHASTER, mobile genetic elements such as prophages, phage-like proteins and pathogenicity islands were located and annotated in each genome. Analysis of the TW20 genome revealed the presence of five prophages, phage-like proteins and/or pathogenicity islands, including ϕSa1, ϕSa3, ϕSPβ-like, SPβ-like proteins, SaPI1. ϕSa1 has been re-classified as ϕSa6 due to both the nature of its integrase, as well as the location of integration (Kahankova et al., 2010; Hyman et al., 2012). The genome of CMRSA6 contains all five of those mobile genetic elements (ϕSa6, ϕSa3, ϕSPβ-like, SPβ-like proteins, SaPI1), while the genome of CMRSA3 contains three (ϕSa6, ϕSa3, SaPI1), and the genome of M92 contains five (ϕSa5, ϕSa3, ϕSPβ-like, SPβ-like proteins, SaPI1) (**Figure 6A**). With some exceptions, the mobile elements were generally quite similar from strain to strain.

ϕSa6 was found in the genomes of TW20, CMRSA6 and CMRSA3, while a very similar phage, ϕSa5, was found in the genome of M92. The integration site for ϕSa5 is located on the opposite side of the M92 genome (near 2000 kb) as the integration site for ϕSa6 in the other three genomes (near 400 kb) (**Figure 6A**). ϕSa5 also differs in that it is inserted in the opposite orientation as compared to ϕSa6 (**Figure 6B**). The same prophage attachment sequences were identified at the left and right extremities of ϕSa6 in TW20, CMRSA6 and CMRSA3 (attL/attR: AAAAAAGGGCAGA), however multiple attachment sequences were identified at each of the extremities of ϕSa5 in M92 (Supplementary Table 1). These include an external sequence pair (attR2/attL2: CTTTTTAAAATTA), and an internal sequence pair (attR3/attL3: TAATTTAGTTAT). This finding suggests that ϕSa5 of M92 may be a composite of two prophages, possibly created by the incomplete excision of one phage, followed by insertion of a second phage. A comparison of ϕSa6 from TW20, CMRSA6, and CMRSA3 revealed that they are highly similar in their protein content (**Figure 6B** and Supplementary Table 1). While ϕSa5 is similar to ϕSa6 in a proportion of the proteins, it differs in the integrase, some of the DNA metabolism proteins, and in the proteins involved with the portal, head, and tail. ϕSa5 also contains 12 phage related proteins between attR2 and attR3 which are not present in ϕSa6. Despite their similarities and differences, it is important to note that ϕSa6 and ϕSa5 are not carrying any known virulence factors which could be responsible for the differential virulence noted among these four strains.

A second prophage, ϕSa3, was found in the genomes of all four strains (TW20, CMRSA6, CMRSA3, and M92) (**Figure 6A**), present at the same location (near 2120 kb) in each strain, with the identical attachment sites (attL/R: AAGTTGCAACAC) identified in TW20, CMRSA6, and CMRSA3 (**Figures 6A,B** and Supplementary Table 2). M92, on the other hand, shares an identical attL sequence with the other three strains, however, it is located internal to alternate attachment sequences (attL2/attR2: AAAAATAATTAG). Once again, this finding suggests the possible role of incomplete excision of a previous prophage, followed by insertion of a new one, creating this composite phage in M92. ϕSa3 in strains TW20 and CMRSA6 are nearly identical, differing primarily in two hypothetical proteins. ϕSa3 of CMRSA3 is also very similar to the corresponding phage in TW20 and CMRSA6, once again differing primarily in hypothetical proteins, as well as in some of the DNA metabolism related proteins (Supplementary Table 2). ϕSa3 of M92, in contrast, is significantly different than ϕSa3 of the other strains. While it shares homology in the integrase and some tail, lysis and virulence associate proteins, it differs significantly in most of the proteins associated with DNA metabolism, portal and head proteins, as well as with some of the tail associated proteins (**Figure 6B**). The genes corresponding to virulence in ϕSa3 include staphylokinase, enterotoxin A, chemotaxis-inhibiting protein (CHIPS), and staphylococcal complement inhibitor (SCIN).

Staphylokinase (SATW20_19380) is present in all four strains, but the gene in CMRSA3 only shares 97% sequence homology with the genes in the other strains. Staphylokinase interacts with host proteins, including alpha-defensins (bactericidal peptides of human neutrophils) and plasminogen (Bokarewa et al., 2006). Binding of staphylokinase to alpha-defensins abolishes their activity, thereby protecting the bacteria from the human innate immune system. Interaction of staphylokinase with plasminogen, on the other hand, forms active plasmin, a proteolytic enzyme that enables bacterial penetration into the surrounding tissues (Bokarewa et al., 2006). Enterotoxin A (SATW20_19410), by contrast, is present in TW20 and CMRSA6, but absent in CMRSA3 and M92. Enterotoxins, including enterotoxin A, are notable virulence factors associated with *S. aureus* and have been implicated in toxic-shock-like syndrome and food poisoning, as well as acting as super-antigens that stimulate T-cell proliferation (Ortega et al., 2010). Finally, the gene for SCIN is present in ϕSa3 of all four strains (SATW20_19360 of TW20) while the gene for CHIPS is only found in CMRSA3. SCIN of M92 differs slightly from the other strains in that it contains a Leu80Gln substitution, but the role of this substitution in terms of protein function is unknown. SCIN inhibits central complement convertase, which reduces phagocytosis of the opsonized organism, blocking all downstream effector functions (Rooijakkers et al., 2007). CHIPS binds to the receptors for C5a and *N*-formyl peptides, reducing leukocyte recruitment (de Haas et al., 2004) and reducing bacterial killing.

A third mobile genetic element found in the genome of these ST239 isolates is the ϕSPβ-like prophage. It is 127.2 kb and integrated near 2200 kb in the genome (**Figure 6A**). ϕSPβ-like was detected in TW20, CMRSA6 and M92, but is absent from CMRSA3. The attL/R sequences (ATTATTATAATT) were identified at both ends of the prophage, including two attL sites located in close proximity to each other (**Figure 6B** and Supplementary Table 3). The phage in all three strains is nearly identical, with very minor variations in a few hypothetical proteins. ϕSPβ-like prophage is a large phage and exhibits similarity with the ϕSPβ-like region of *S. epidermidis* RP62a (Gill et al., 2005). It contains genes associated with aminoglycoside resistance, making the treatment of infection difficult (Holden et al., 2010). This phage also contains genes which may have a role in persistence of the organism in hospital settings (Holden et al., 2010). Since TW20, CMRSA6, and M92 belong to ST239, a major hospital associated MLST type, this phage likely plays an important role in their maintenance in the strong antibiotic selective pressures found in hospital environments. The ϕSPβ-like phage does not show similarity with any other *S. aureus* prophage and has been shown to be present only in epidemic ST239 strains (Xia and Wolz, 2014). CMRSA3, although closely related, belongs to ST241, which could explain why it is devoid of this prophage. This would, in turn, possibly explain why CMRSA3 (originally one of 10 epidemic MRSA strains) had virtually disappeared in Canada after 1997, being replaced by another closely related epidemic strain CMRSA6 (Christianson et al., 2007). In addition to ϕSPβ-like prophage, the genomes of TW20, CMRSA6, and M92 also carry ϕSPβ-like proteins near 1400 kb on their genomes (**Figure 6A**). These proteins show similarity to proteins in the ϕSPβ-like prophage (**Figure 6B** and Supplementary Table 4) and likely represent prophage remnants. The ϕSPβ-like proteins do not appear to contribute to virulence of the strains as no genes related to virulence were detected.

The final mobile genetic element detected in the genomes of TW20, CMRSA6, CMRSA3 and M92, is a pathogenicity island, SaPI1. It is located near 960 kb and contains attL/attR sequences (TTGAAAATAAAA) on each end (**Figures 6A,B** and Supplementary Table 5). The pathogenicity island proteins are nearly identical in all four strains. SaPI1 contains genes coding for enterotoxins K and Q (SATW20_08900 and SATW20_08910 of TW20 respectively), which are reported to play an important role in staphylococcal diseases (like food poisoning), as mentioned earlier.

### Plasmids Identified in TW20 and CMRSA3

Extrachromosomal genetic material often carries resistance determinants and genes essential for virulence and pathogenicity of an organism. Among TW20, CMRSA6, CMRSA3 and M92, only TW20 and CMRSA3 were found to carry plasmids. The plasmids of TW20, TW20_1, and TW20_2 are 29.5 and 3 kb respectively (Holden et al., 2010). TW20_1 carries important resistance determinants, including the gene coding for QacA, an antiseptic resistance protein conferring resistance to quaternary ammonium salts, cationic biocides and diamidines. TW20_1 also carries the *mer* and *cad* operons, containing genes coding for resistance to mercury (MerA, mercuric reductase) and cadmium (CadA, a cadmium transporting ATPase and CadD, cadmium resistance protein), respectively. The second plasmid carried by TW20, TW20_2, is approximately 3 kb and codes only for a replication origin and hypothetical protein. This plasmid is unlikely to contribute to virulence of the strain.

The plasmid, pCMRSA3 carried by CMRSA3 is 27 kb long and has components resembling pTW20_1 of TW20 and pZ172 of *S. aureus* subsp. *aureus* Z712. The arrangement of genes within these plasmids varies, however, the complementary regions among them share 99% homology. Like pTW20_1 and pZ172, pCMRSA3 carries genes for antiseptic resistance (*qacA*), mercury resistance (*merA*), and cadmium resistance (*cadD* and *cadA*).

Biocides and quaternary ammonium compounds are used as antiseptics on body surfaces, as well as disinfectants on equipment and surfaces in many environments such as hospitals or farms. These compounds are also being used to improve hygiene, while some of the heavy metals that are relatively non-toxic to mammalian tissue are used as antimicrobial coatings and wound dressings (Wales and Davies, 2015). Resistance to these agents provides survival benefits to the organisms.

### Antibiotic Resistance Profiles and Other Genes Contributing to Virulence

Resistance to multiple types of antibiotics plays an important role in the ability of an organism to survive, particularly within hospital environments where there is significant antibiotic selective pressure. The antibiotic resistance profiles of CMRSA6, CMRSA3, and M92 reveal that they are resistant to the majority of the antibiotics used in hospitals, a characteristic typical of HA-MRSA strains (**Table 1**). Their resistance profiles are nearly identical, with the exception of trimethoprim/sulfamethoxazole (where CMRSA6 and CMRSA3 are resistant, while M92 is susceptible) and gentamicin (where CMRSA6 and M92 are resistant, while CMRSA3 is susceptible). As discussed earlier, M92 lacks *dfrG*, which codes for the trimethoprim-resistant dihydrofolate reductase, making it susceptible to trimethoprim (**Figure 5A** and **Table 1**). CMRSA6 and CMRSA3, in contrast, both have the *dfrG* gene, protecting them from the action of trimethoprim (**Figure 5** and **Table 1**). Since limited data is available from the publication regarding the resistance profile of TW20 (Holden et al., 2010), we could not compare it fully to CMRSA6, CMRSA3, and M92. However, the available information does indicate that TW20’s resistance pattern is consistent with that of a HA-MRSA, as it is resistant to the core antibiotics used in hospitals (including trimethoprim and gentamicin), in addition to β-lactams.

Surface anchored proteins with LPxTG motif bind host molecules and have been shown to be present in only 7% of ST239 strains. The presence of a LPxTG motif surface-anchored protein in TW20 (*sasX*; SATW20_21850) is proposed to be linked to its increased virulence and invasive capacity (Holden et al., 2010). Interestingly, this protein is also detected in the genomes of moderately virulent CMRSA6 and avirulent M92, suggesting that it likely plays a minor role in the augmented virulence of TW20. The gene was not detected in CMRSA3, which belongs to ST241, correlating well with findings that orthologs of this protein have not been detected in the sequenced genomes of *S. aureus* other than ST239 (Holden et al., 2010).

DNA gyrase subunit A of TW20 has a point mutation resulting in the substitution of Ser to Leu at position 84, which is in contrast to the majority of *S. aureus* strains that contain serine at that position. Studies have demonstrated that the Ser84Leu substitution is associated with resistance to quinolones, which may promote survival in the hospital environment. This point mutation was detected in all four of the ST239 isolates analyzed here.

Isoleucyl-tRNA synthetase in TW20 contains a Val588Phe substitution, which has been shown to confer chromosomal low-level mupirocin resistance. This substitution was not detected in CMRSA6, CMRSA3, or M92. CMRSA3 did, however, have an isoleucine instead of phenylalanine at position 581, the significance of which is unknown.

## Conclusion

Comparative genomic analysis of TW20, CMRSA6, CMRSA3, and M92 reveals remarkable similarities. Pulsotypes and SNP WGS phylogenetic cladograms of all four strains show that they cluster together forming a genomicly closely-related ST239 sub-lineage. While TW20 is positive for every genetic trait put forth as a possible contributor to virulence, CMRSA6, CMRSA3, and M92 showed variations in terms of carrying these traits (**Table 2**).

**Table 2:** Summary of the genetic variation among the ST239 sub-lineage strains.

Genomic component	ST239 strain			
	TW20	CMRSA6	CMRSA3	M92
*spa*	+	+	+	-
*lpl*	Intact	Disrupted	Disrupted	Disrupted
*cta*	Intact	IS256 insertion	Intact	Intact
*dfrG*^†^	+	+	+ (two copies)	-
*gyrA*^†^	Ser84Leu	Ser84Leu	Ser84Leu	Ser84Leu
Isoleucyl-tRNA synthetase^†^	Val588Phe	Val	Isl581Phe	Val
ϕSa6^†^ - No proteins related to virulence	+	+^∗^	+^∗∗^	-
ϕSa5 - No proteins related to virulence	-	-	-	+^∗∗∗^
ϕSa3^†^	+	+^∗^	+^∗^	+^∗∗∗^
-Staphylokinase	+	+	+	+
-Enterotoxin A	+	+	-	-
-SCIN	+	+	+	+
-CHIPS	-	-	+	-
ϕSPβ-like^†^ - Resistance and persistence genes	+	+^∗^	-	+^∗^
- *sasX* (LPxTG protein)^†^	+	+	-	+
ϕSPβ-like proteins - No proteins related to virulence	+	+^∗^	-	+^∗^
SaPI1^†^	+	+^∗^	+^∗^	+^∗^
-Enterotoxin K	+	+	+	+
-Enterotoxin Q	+	+	+	+
Major plasmid^†^	pTW20_1	-	pCMRSA3_1	-
-QacA	+		+	
-Mercury resistance	+		+	
-Cadmium resistance	+		+	
Minor plasmid^†^ - No proteins related to virulence	pTW20_2	-	-	-

*^†^Loci/elements proposed by Holden et al. (2010) to contribute to the virulence of TW20. ^∗^ Nearly identical to the region in TW20 (≥99% homology). ^∗∗^ Very similar to the region in TW20, with minor variations in protein content (90% homology). ^∗∗∗^ Significantly different from the region in TW20, with small regions of homology (≤50% overall homology).*

The major components differing among these strains are staphylococcal protein A (SpA) and the lipoprotein-like proteins (Lpl). The SpA of TW20, CMRSA6 and CMRSA3 is identical, while M92 is likely devoid of a functional *spa* gene encoded protein (**Table 2**). Similarly, the Lpl transcribed by TW20 has recently been shown to play a vital role in the pathogenesis of *S. aureus* species, but is disrupted in CMRSA6, CMRSA3, and M92.Mobile genetic elements may also play a role in the virulence of these strains. Three prophages (ϕSa6, ϕSa3, and ϕSPβ-like), a pathogenicity island (SaPI1) and two plasmids were located in strain TW20, and are present with variations in one or more of the other three strains (**Table 2**). Despite similar mobile element carriage, it is important to highlight the fact that even if similar virulence factors are present, these mobile elements are not identical; they have slight variations in their content with respect to hypothetical proteins of unknown function, any of which could play a significant role in the pathogenesis of the strain.

Further studies are needed to examine each of the genomic components brought forth in this study, with the goal of determining their exact contribution to the virulence and pathogenesis of TW20, CMRSA6, CMRSA3, and M92. None of these components exists in isolation, meaning the full virulence of *S. aureus* ST239 likely results from the sum of, and interplay between multiple factors.

## Author Contributions

KZ conceived, designed, and supervised the work. J-AM and AK performed the experiments and analyzed data. JC provided the clinical information. SL and KZ structured and drafted the manuscript. J-AM, JC, and KZ reviewed and edited the manuscript.

## Conflict of Interest Statement

The authors declare that the research was conducted in the absence of any commercial or financial relationships that could be construed as a potential conflict of interest. The reviewer BM and handling Editor declared their shared affiliation.
