# Supplementary material for: Genomic Comparison of Highly Virulent, Moderately Virulent, and Avirulent Strains From a Genetically Closely-Related MRSA ST239 Sub-lineage Provides Insights Into Pathogenesis
Source: Front Microbiol. 2018 Jul 10;9:1531. doi: 10.3389/fmicb.2018.01531 (PMC6048232; doi:10.3389/fmicb.2018.01531)
Supplement: Supplementary file 1 [file Table_1.DOCX]

**Suppl.Table 1.** φSa6 and φSa5 component comparison based on PHASTER annotation.

|  | **φSA6** | | | **φSA5** |
| --- | --- | --- | --- | --- |
| **Gene Product** | **TW20** | **CMRSA6** | **CMRSA3** | **M92** |
| **attR-2 (M92) CTTTTTAAAATTA** | - | - | - | + |
| hypothetical protein (gi100164) | - | - | - | + |
| integrase (gi118430725) | - | - | - | + |
| excisionase (gi966198844) | - | - | - | + |
| hypothetical protein (gi966198845) | - | - | - | + |
| hypothetical protein (gi966198846) | - | - | - | + |
| repressor (gi966198847) | - | - | - | + |
| repressor (gi966198848) | - | - | - | + |
| hypothetical protein (gi966198849) | - | - | - | + |
| hypothetical protein (gi966198850) | - | - | - | + |
| Phage anti-repressor protein (gi66395232) | - | - | - | + |
| ORF091 (gi66396257) | - | - | - | + |
| ORF058 (gi66396241) | - | - | - | + |
| **attR-3 (M92) TAATTTAGTTAT** | - | - | - | + |
| ORF071 (gi66396392) | - | - | - | + |
| hypothetical protein (gi966198854) | - | - | - | + |
| hypothetical protein (gi588498258) | - | - | - | + |
| hypothetical protein (gi30043975) | - | - | - | + |
| hypothetical protein (gi156603902) | - | - | - | + |
| **attL AAAAAAGGGCAGA** | + | + | + | - |
| integrase (gi966198843) | + | + | + | - |
| excisionase (gi971755111) | + | + | + | - |
| ORF013 (gi66395383) | + | + | + | - |
| hypothetical protein (gi526244875) | + | + | + | - |
| hypothetical protein (gi526244876) | + | + | + | - |
| putative Cro/CI family transcriptional regulator (gi526244877) | + | + | + | - |
| putative Cro protein (gi526244878) | + | + | + | - |
| anti-repressor KilAC domain protein (gi526244879) | + | + | + | - |
| superantigen-encoding pathogenicity island protein (gi971755515) | + | + | + | - |
| 77ORF065 (gi41189566) | + | + | - | - |
| hypothetical protein (gi209363565) | + | + | + | - |
| 77ORF102 (gi41189577) | + | + | + | - |
| 77ORF043 (gi41189554) | + | + | + | - |
| hypothetical protein (gi526178042) | + | + | + | + |
| recombination protein (gi744692799) | + | + | + | + |
| hypothetical protein (gi30043971) | + | + | + | + |
| single-stranded DNA binding protein (gi744692798) | + | + | + | + |
| replication protein (gi744692797) | + | + | + | + |
| hypothetical protein (gi526003559) | + | + | + | + |
| DNA replication, recombination, and repair (gi744692796) | + | + | + | + |
| hypothetical protein (gi30043966) | + | + | + | + |
| hypothetical protein (gi30043965) | + | + | + | + |
| hypothetical protein (gi30043964) | + | + | + | + |
| ORF036 (gi66396442) | + | + | + | + |
| hypothetical protein (gi526244898) | + | + | + | - |
| hypothetical protein (gi526244899) | + | + | + | - |
| hypothetical protein (gi966198872) | - | - | - | + |
| ORF053 (gi66394917) | - | - | - | + |
| hypothetical protein (gi526244900) | + | + | + | + |
| putative dUTP diphosphatase (gi526244901) | + | + | + | + |
| hypothetical protein (gi66396173) | + | + | + | + |
| 77ORF072 (gi41189571) | + | + | + | - |
| hypothetical protein (gi966198879) | - | - | - | + |
| hypothetical protein (gi966198880) | + | + | + | + |
| transcriptional activator RinB (gi526244904) | + | + | + | - |
| hypothetical protein (gi526244905) | + | + | + | - |
| ORF118 (gi66396262) | + | + | + | - |
| transcriptional activator RinA (gi526244906) | + | + | + | - |
| terminase small subunit (gi526244907) | + | + | + | - |
| PBSX family terminase large subunit (gi526244908) | + | + | + | - |
| SPP1 family portal protein (gi526244909) | + | + | + | - |
| hypothetical protein (gi966198881) | - | - | - | + |
| transcriptional activator RinB (gi966198882) | - | - | - | + |
| hypothetical protein (gi966198883) | - | - | - | + |
| terminase small subunit (gi966198884) | - | - | - | + |
| terminase large subunit (gi966198885) | - | - | - | + |
| SPP1 family portal protein (gi966198886) | - | - | - | + |
| head morphogenesis protein (gi966198887) | + | + | + | + |
| hypothetical protein (gi966198888) | + | + | + | + |
| minor structural protein GP20 (gi526244911) | + | + | + | - |
| major capsid protein (gi526244912) | + | + | + | - |
| rho termination factor domain-containing protein (gi526244913) | + | + | + | - |
| phage gp6-like head-tail connector protein (gi526244914) | + | + | + | - |
| putative phage head-tail adaptor (gi526244915) | + | + | + | - |
| hypothetical protein (gi526244916) | + | + | + | - |
| hypothetical protein (gi526244917) | + | + | + | - |
| putative major tail protein (gi526244918) | + | + | + | - |
| putative tail assembly protein (gi526244919) | + | + | + | - |
| ORF045 (gi66394712) | + | + | + | - |
| putative tape measure protein (gi526244921) | + | + | + | - |
| hypothetical protein (gi100048) | + | + | + | + |
| putative minor structural protein (gi526244923) | + | + | + | - |
| ORF002 (gi66395887) | + | + | + | - |
| tail protein (gi971765365) | + | + | + | - |
| hypothetical protein (gi966198889) | - | - | - | + |
| major capsid protein (gi966198890) | - | - | - | + |
| hypothetical protein (gi966198891) | - | - | - | + |
| putative DNA packaging protein (gi966198892) | - | - | - | + |
| putative SPP1 family head-tail adaptor (gi966198893) | - | - | - | + |
| putative head-tail component (gi966198894) | - | - | - | + |
| minor tail protein (gi966198895) | - | - | - | + |
| major tail protein (gi966198896) | - | - | - | + |
| hypothetical protein (gi966198897) | - | - | - | + |
| hypothetical protein (gi966198898) | - | - | - | + |
| tape measure protein (gi971755340) | - | - | - | + |
| hypothetical protein (gi100048) | - | - | - | + |
| Prophage endopeptidase tail (gi66396116) | - | - | - | + |
| hypothetical protein (gi966198902) | - | - | - | + |
| hypothetical protein (gi966198903) | - | - | - | + |
| hypothetical protein (gi971755466) | + | + | + | + |
| hypothetical protein (gi971755465) | + | + | + | + |
| hypothetical protein (gi526244928) | + | + | + | - |
| hypothetical protein (gi966198906) | - | - | - | + |
| tail tip protein (gi162290169) | + | + | + | + |
| tail fiber (gi304443261) | + | + | + | + |
| ORF035 (gi66396148) | + | + | + | + |
| putative holin protein (gi162290172) | + | + | + | + |
| endolysin; PP_00395 (gi162290173) | + | + | + | - |
| ORF007 (gi66396198) | - | - | - | + |
| hypothetical protein (gi526244934) | - | - | - | + |
| **attR AAAAAAGGGCAGA** | + | + | + | - |
| **attL-3 TAATTTAGTTAT** | - | - | - | + |
| **attL-2 CTTTTTAAAATTA** | - | - | - | + |
