# Supplementary material for: Genomic Comparison of Highly Virulent, Moderately Virulent, and Avirulent Strains From a Genetically Closely-Related MRSA ST239 Sub-lineage Provides Insights Into Pathogenesis
Source: Front Microbiol. 2018 Jul 10;9:1531. doi: 10.3389/fmicb.2018.01531 (PMC6048232; doi:10.3389/fmicb.2018.01531)
Supplement: Supplementary file 2 [file Table_2.DOCX]

**Suppl.Table 2.** φSa3 component comparison based on PHASTER annotation.

| **Gene Product** | **TW20** | **CMRSA6** | **CMRSA3** | **M92** |
| --- | --- | --- | --- | --- |
| **attL-2 (M92) AAAAATAATTAG** | - | - | - | + |
| **attL AAGTTGCAACAC** | + | + | + | + |
| hypothetical protein (gi118725118) | + | + | - | - |
| hypothetical protein (gi30043926) | - | - | + | - |
| hypothetical protein (gi526003548) | + | + | + | + |
| complement inhibitor (gi526003581) | + | + | + | + |
| chemotaxis-inhibiting protein CHIPS (gi118725116) | - | - | + | - |
| truncated amidase (gi118725115) | + | + | + | - |
| staphylokinase (gi526003580) | + | + | - | - |
| staphylokinase precursor (gi118725114) | - | - | + | - |
| amidase (gi118725113) | + | + | + | - |
| holin (gi118725112) | + | + | - | - |
| enterotoxin type A precursor (gi118725111) | + | + | - | - |
| hypothetical protein (gi156603943) | + | + | - | - |
| hypothetical protein (gi30043939) | + | + | - | - |
| transposase mutator family (gi985761243) | - | - | - | + |
| amidase (gi538397809) | - | - | - | + |
| staphylokinase (gi29028715) | - | - | - | + |
| autolysin (gi526003579) | - | - | - | + |
| holin (gi526003578) | - | - | - | + |
| hypothetical protein (gi526003547) | - | - | - | + |
| phi PVL ORF 22-like protein (gi526003546) | - | - | - | + |
| hypothetical protein (gi526003545) | + | + | - | + |
| holin (gi257136422) | - | - | + | - |
| phi PVL ORF 17 homologue (gi9635733) | - | - | + | - |
| phi PVL ORF 22 homologue (gi9635732) | - | - | + | - |
| hypothetical protein (gi118725108) | - | - | + | - |
| phage minor structural protein (gi118725107) | + | + | + | - |
| hypothetical protein (gi118725106) | + | + | + | - |
| phage tail tape measure protein (gi118725105) | + | + | + | - |
| hypothetical protein (gi118725104) | + | + | + | - |
| hypothetical protein (gi118725103) | + | + | + | - |
| hypothetical protein (gi118725102) | + | + | + | - |
| tail superfamily protein (gi588498291) | + | + | + | - |
| hypothetical protein (gi118725100) | + | + | + | - |
| hypothetical protein (gi118725099) | + | + | + | - |
| head-tail adaptor (gi744692788) | + | + | + | - |
| hypothetical protein (gi118725097) | + | + | + | - |
| hypothetical protein (gi118725096) | + | + | + | - |
| hypothetical protein (gi118725095) | + | + | + | - |
| protease/scaffold protein (gi257136405) | + | + | + | - |
| phage portal protein (gi118725093) | + | + | + | - |
| phage terminase (gi118725092) | + | + | + | - |
| hypothetical protein (gi118725091) | + | + | + | - |
| hypothetical protein (gi118725090) | + | + | + | - |
| hypothetical protein (gi118725089) | + | + | + | - |
| hypothetical protein (gi118725088) | + | + | + | - |
| hypothetical protein (gi118725087) | + | + | + | - |
| hypothetical protein (gi744692815) | + | + | + | - |
| hypothetical protein (gi66396173) | + | + | + | - |
| phi ETA orf 34-like protein (gi29028691) | + | + | + | - |
| hypothetical protein (gi257136398) | + | + | + | - |
| hypothetical protein (gi971749899) | + | + | + | - |
| hypothetical protein (gi526178052) | + | + | + | - |
| hypothetical protein (gi526003593) | + | + | + | - |
| hypothetical protein (gi30043964) | + | - | - | - |
| hypothetical protein (gi971750178) | - | + | - | - |
| hypothetical protein (gi971750178) | - | - | + | - |
| hypothetical protein (gi30043965) | + | + | + | - |
| hypothetical protein (gi118725079) | + | + | + | - |
| DNA replication, recombination, and repair (gi744692796) | + | + | + | - |
| hypothetical protein (gi526003559) | + | + | + | - |
| replication protein (gi744692797) | + | + | + | - |
| single-stranded DNA binding protein (gi744692798) | + | + | + | - |
| hypothetical protein (gi118725074) | + | + | + | - |
| recombination protein (gi744692799) | + | + | + | - |
| hypothetical protein (gi118725072) | + | + | + | - |
| hypothetical protein (gi118725071) | + | - | - | - |
| phi PVL orf 39-like protein (gi29028574) | + | + | + | - |
| ORF047(gi66396451) | + | + | + | - |
| hypothetical protein (gi118725067) | + | + | + | - |
| hypothetical protein (gi118725066) | + | + | + | - |
| hypothetical protein (gi118725065) | + | + | + | - |
| hypothetical protein (gi588498253) | + | + | + | - |
| hypothetical protein (gi118725064) | + | + | + | - |
| anti-repressor (gi9635199) | + | + | + | - |
| hypothetical protein (gi118725062) | + | + | + | - |
| transcriptional regulato (gi118725060) | + | + | + | - |
| putative repressor (gi118725059) | + | + | + | - |
| probable ATP-dependent helicase (gi118725058) | + | + | + | - |
| hypothetical protein (gi118725057) | + | + | - | - |
| hypothetical protein (gi118725056) | + | + | + | - |
| putative lipoprotein (gi118725055) | + | + | + | - |
| minor structural protein (gi526003577) | - | - | - | + |
| hypothetical protein (gi526003544) | - | - | - | + |
| tail length tape-measure protein (gi538397810) | - | - | - | + |
| hypothetical protein (gi526003574) | - | - | - | + |
| hypothetical protein (gi526003573) | - | - | - | + |
| putative major tail protein, Phi13 family (gi526003603) | - | - | - | + |
| Phi PVL orf 12-like protein (gi526003572) | - | - | - | + |
| HK97 GP10 family phage protein (gi526003602) | - | - | - | + |
| putative phage head tail adapter (gi156603930) | - | - | - | + |
| hypothetical protein (gi526003571) | - | - | - | + |
| hypothetical protein (gi526003570) | - | - | - | + |
| capsid protein (gi526003601) | - | - | - | + |
| putative prohead protease (gi526003600) | - | - | - | + |
| portal protein (gi239507400) | - | - | - | + |
| putative terminase large subunit (gi156603924) | - | - | - | + |
| phi PVL ORF 2 homologue (gi9635715) | - | - | - | + |
| putative terminase small subunit (gi526003597) | - | - | - | + |
| endonuclease (gi526003569) | - | - | - | + |
| hypothetical protein (gi156603920) | - | - | - | + |
| hypothetical protein (gi526003567) | - | - | - | + |
| hypothetical protein (gi526003596) | - | - | - | + |
| RinB (gi41189581) | - | - | - | + |
| hypothetical protein (gi66395997) | - | - | - | + |
| ORF061 (gi66395342) | - | - | - | + |
| hypothetical protein (gi509140973) | - | - | - | + |
| hypothetical protein (gi215401202) | - | - | - | + |
| hypothetical protein (gi526003563) | - | - | - | + |
| hypothetical protein (gi526003594) | - | - | - | + |
| ORF053 (gi66394917) | - | - | - | + |
| YopX (gi41189548) | - | - | - | + |
| hypothetical protein (gi509139902) | - | - | - | + |
| hypothetical protein (gi966198870) | - | - | - | + |
| PVL orf 51-like protein (gi29028634) | - | - | - | + |
| hypothetical protein (gi66395418) | - | - | - | + |
| ORF041 (gi66395406) | - | - | - | + |
| hypothetical protein (gi66395431) | - | - | - | + |
| RusA (gi66395400) | - | - | - | + |
| hypothetical protein (gi66395422) | - | - | - | + |
| ORF101 (gi66395362) | - | - | - | + |
| hypothetical protein (gi100018) | - | - | - | + |
| hypothetical protein (gi304443284) | - | - | - | + |
| hypothetical protein (gi100016) | - | - | - | + |
| Putative HNHc nuclease (gi66396352) | - | - | - | + |
| single strand DNA binding protein (gi257136374) | - | - | - | + |
| hypothetical protein (gi66395393) | - | - | - | + |
| hypothetical protein (gi41189565) | - | - | - | + |
| phi PVL orf 39-like protein (gi526003586) | - | - | - | + |
| hypothetical protein (gi526178039) | - | - | - | + |
| hypothetical protein (gi209363565) | - | - | - | + |
| anti-repressor (gi971742289) | - | - | - | + |
| ORF049 (gi66395555) | - | - | - | + |
| ORF073 (gi66395425) | - | - | - | + |
| cI-like repressor (gi29028568) | - | - | - | + |
| hypothetical protein (gi209363556) | - | - | - | + |
| hypothetical protein (gi526003551) | - | - | - | + |
| phi PVL ORF 30-like protein (gi526003550) | - | - | - | + |
| **attR AAGTTGCAACAC** | + | + | + |  |
| **attR-2 (M92) AAAAATAATTAG** |  |  |  |  |
| integrase (gi118725054) | + | + | + | - |
