# Supplementary material for: Genomic Comparison of Highly Virulent, Moderately Virulent, and Avirulent Strains From a Genetically Closely-Related MRSA ST239 Sub-lineage Provides Insights Into Pathogenesis
Source: Front Microbiol. 2018 Jul 10;9:1531. doi: 10.3389/fmicb.2018.01531 (PMC6048232; doi:10.3389/fmicb.2018.01531)
Supplement: Supplementary file 3 [file Table_3.DOCX]

**Suppl.Table 3.** φSPβ-like component comparison based on PHASTER annotation.

| **Gene product** | **TW20** | **CMRSA6** | **CMRSA3** | **M92** |
| --- | --- | --- | --- | --- |
| **attL ATTATTATAATT** | **+** | **+** |  | **+** |
| **attL ATTATTATAATT** | **+** | **+** |  | **+** |
| recombinase (gi985761159) | **+** | **+** |  | **+** |
| Myb-like DNA-binding domain protein (gi985761160) | **+** | **+** |  | **+** |
| hypothetical protein | **+** | **+** |  | **+** |
| endodeoxyribonuclease RusA (gi985761161) | **+** | **+** |  | **+** |
| hypothetical protein (gi985761162) | **+** | **+** |  | **+** |
| phosphoribosyl-ATP pyrophosphohydrolase (gi985761163) | **+** | **+** |  | **+** |
| hypothetical protein (gi985761164) | **+** | **+** |  | **+** |
| thioredoxin; PP_02064 (gi985761165) | **+** | **+** |  | **+** |
| hypothetical protein (gi985761166) | **+** | **+** |  | **+** |
| ribonucleoside-diphosphate reductase subunit beta (gi985761167) | **+** | **+** |  | **+** |
| Ribonucleotide reductase and Pyruvate formate lyase (gi66391367) | **+** | **+** |  | **+** |
| ribonucleoside-diphosphate reductase subunit alpha 2 (gi985761169) | **+** | **+** |  | **+** |
| hypothetical protein (gi985761170) | **+** | **+** |  | **+** |
| putative NrdI-like protein (gi985761171) | **+** | **+** |  | **+** |
| hypothetical protein (gi985761172) | **+** | **+** |  | **+** |
| hypothetical protein (gi985761173) | **+** | **+** |  | **+** |
| hypothetical protein (gi985761174) | **+** | **+** |  | **+** |
| hypothetical protein (gi985761175) | **+** | **+** |  | **+** |
| hypothetical protein (gi985761176) | **+** | **+** |  | **+** |
| hypothetical protein (gi985761177) | **+** | **+** |  | **+** |
| hypothetical protein (gi985761178) | **+** | **+** |  | **+** |
| modification methylase HhaI (gi985761179) | **+** | **+** |  | **+** |
| tRNA (cmo5U34)-methyltransferase (gi985761180) | **+** | **+** |  | **+** |
| hypothetical protein (gi985761181) | **+** | **+** |  | **+** |
| nucleoside 2-deoxyribosyltransferase (gi985761182) | **+** | **+** |  | **+** |
| hypothetical protein (gi985761183) | **+** | **+** |  | **+** |
| hypothetical protein (gi985761184) | **+** | **+** |  | **+** |
| hypothetical protein (gi985761185) | **+** | **+** |  | **+** |
| hypothetical protein (gi985761186) | **+** | **+** |  | **+** |
| hypothetical protein (gi985761187) | **+** | **+** |  | **+** |
| hypothetical protein (gi985761188) | **+** | **+** |  | **+** |
| hypothetical protein (gi985761189) | **+** | **+** |  | **+** |
| DNA ligase (gi985761190) | **+** | **+** |  | **+** |
| hypothetical protein (gi985761191) | **+** | **+** |  | **+** |
| hypothetical protein (gi985761192) | **+** | **+** |  | **+** |
| DNA polymerase III subunit alpha (gi985761193) | **+** | **+** |  | **+** |
| DNA polymerase III subunit alpha (gi985761195) | **+** | **+** |  | **+** |
| hypothetical protein (gi985761196) | **-** | **+** |  | **+** |
| hypothetical protein (gi985761197) | **+** | **+** |  | **+** |
| hypothetical protein | **+** | **+** |  | **+** |
| transposase mutator family (gi985761243) | **+** | **+** |  | **+** |
| bifunctional AAC/APH (gi985761240) | **+** | **+** |  | **+** |
| ribosomal-protein-alanine N-acetyltransferase (gi985761241) | **+** | **+** |  | **+** |
| hypothetical protein | **+** | **+** |  | **+** |
| hypothetical protein | **+** | **+** |  | **+** |
| hypothetical protein | **+** | **+** |  | **+** |
| hypothetical protein | **+** | **+** |  | **+** |
| hypothetical protein | **+** | **-** |  | **+** |
| hypothetical protein | **-** | **-** |  | **+** |
| hypothetical protein (gi985761201) | **+** | **+** |  | **+** |
| calcineurin-like phosphoesterase superfamily domain protein (gi985761202) | **+** | **+** |  | **+** |
| hypothetical protein (gi985761203) | **+** | **+** |  | **+** |
| hypothetical protein (gi985761204) | **+** | **+** |  | **+** |
| hypothetical protein (gi985761205) | **+** | **+** |  | **+** |
| Fic/DOC family protein (gi985761206) | **+** | **+** |  | **+** |
| hypothetical protein (gi985761207) | **+** | **+** |  | **+** |
| hypothetical protein (gi985761208) | **+** | **+** |  | **+** |
| hypothetical protein (gi985761209) | **+** | **+** |  | **+** |
| hypothetical protein (gi985761210) | **+** | **+** |  | **+** |
| diadenosine tetraphosphatase (gi985761211) | **+** | **+** |  | **+** |
| hypothetical protein (gi985761212) | **+** | **+** |  | **+** |
| hypothetical protein (gi985761213) | **+** | **+** |  | **+** |
| hypothetical protein (gi985761214) | **+** | **+** |  | **+** |
| hypothetical protein (gi985761215) | **+** | **+** |  | **+** |
| transcriptional activator RinB (gi985761216) | **+** | **+** |  | **+** |
| hypothetical protein (gi985761217) | **+** | **+** |  | **+** |
| hypothetical protein (gi985761218) | **+** | **+** |  | **+** |
| hypothetical protein (gi985761219) | **+** | **+** |  | **+** |
| hypothetical protein (gi985761220) | **+** | **+** |  | **+** |
| hypothetical protein (gi985761222) | **+** | **+** |  | **+** |
| hypothetical protein | **+** | **+** |  | **+** |
| hypothetical protein (gi985761223) | **+** | **+** |  | **+** |
| hypothetical protein | **+** | **+** |  | **+** |
| hypothetical protein | **+** | **+** |  | **+** |
| hypothetical protein (gi985761225) | **+** | **+** |  | **+** |
| hypothetical protein (gi985761226) | **+** | **+** |  | **+** |
| thermonuclease precursor (gi985761227) | **+** | **+** |  | **+** |
| YopX protein (gi985761228) | **+** | **+** |  | **+** |
| hypothetical protein (gi985761229) | **+** | **+** |  | **+** |
| hypothetical protein (gi971766731) | **+** | **+** |  | **+** |
| hypothetical protein (gi985761230) | **+** | **+** |  | **+** |
| hypothetical protein (gi985761233) | **+** | **+** |  | **+** |
| hypothetical protein (gi985761234) | **+** | **+** |  | **+** |
| hypothetical protein (gi985761235) | **+** | **+** |  | **+** |
| ribonuclease Z (gi985761091) | **+** | **+** |  | **+** |
| single-strand DNA-specific exonuclease YorK (gi985761092) | **+** | **+** |  | **+** |
| hypothetical protein (gi985761093) | **+** | **+** |  | **+** |
| replicative DNA helicase (gi985761094) | **+** | **+** |  | **+** |
| hypothetical protein (gi985761095) | **+** | **+** |  | **+** |
| hypothetical protein (gi985761096) | **+** | **+** |  | **+** |
| hypothetical protein (gi985761097) | **+** | **+** |  | **+** |
| hypothetical protein (gi985761098) | **+** | **+** |  | **+** |
| hypothetical protein (gi985761099) | **+** | **+** |  | **+** |
| hypothetical protein (gi985761100) | **+** | **+** |  | **+** |
| hypothetical protein (gi985761101) | **+** | **+** |  | **+** |
| hypothetical protein (gi985761102) | **+** | **+** |  | **+** |
| site-specific tyrosine recombinase XerC (gi985761103) | **+** | **+** |  | **+** |
| hypothetical protein (gi985761104) | **+** | **+** |  | **+** |
| hypothetical protein (gi985761105) | **+** | **+** |  | **+** |
| hypothetical protein (gi985761106) | **+** | **+** |  | **+** |
| DNA polymerase III subunit beta (gi985761107) | **+** | **+** |  | **+** |
| hypothetical protein (gi985761109) | **+** | **+** |  | **+** |
| antirestriction protein (gi985761110) | **+** | **+** |  | **+** |
| hypothetical protein (gi985761111) | **+** | **+** |  | **+** |
| hypothetical protein (gi985761112) | **+** | **+** |  | **+** |
| hypothetical protein (gi985761113) | **+** | **+** |  | **+** |
| hypothetical protein (gi985761114) | **+** | **+** |  | **+** |
| hypothetical protein (gi985761115) | **+** | **+** |  | **+** |
| hypothetical protein | **+** | **+** |  | **+** |
| hypothetical protein (gi985761116) | **-** | **+** |  | **-** |
| hypothetical protein (gi985761117) | **+** | **+** |  | **+** |
| ATP-dependent RecD-like DNA helicase (gi985761118) | **+** | **+** |  | **+** |
| hypothetical protein (gi985761119) | **+** | **+** |  | **+** |
| DNA-binding protein HU 1 (gi985761120) | **+** | **+** |  | **+** |
| YonK protein (gi985761121) | **+** | **+** |  | **+** |
| hypothetical protein (gi985761122) | **+** | **+** |  | **+** |
| hypothetical protein (gi985761123) | **+** | **+** |  | **+** |
| hypothetical protein (gi985761125) | **+** | **+** |  | **+** |
| hypothetical protein (gi985761126) | **+** | **+** |  | **+** |
| hypothetical protein (gi985761127) | **+** | **+** |  | **+** |
| hypothetical protein (gi985761128) | **+** | **+** |  | **+** |
| hypothetical protein (gi985761130) | **+** | **+** |  | **+** |
| hypothetical protein (gi985761131) | **+** | **+** |  | **+** |
| hypothetical protein (gi985761132) | **+** | **+** |  | **+** |
| hypothetical protein (gi985761133) | **+** | **+** |  | **+** |
| hypothetical protein (gi985761134) | **+** | **+** |  | **+** |
| hypothetical protein (gi985761135) | **+** | **+** |  | **+** |
| hypothetical protein (gi985761136) | **+** | **+** |  | **+** |
| hypothetical protein (gi985761137) | **+** | **+** |  | **+** |
| hypothetical protein (gi985761138) | **+** | **+** |  | **+** |
| hypothetical protein (gi985761139) | **+** | **+** |  | **+** |
| site-specific tyrosine recombinase XerC (gi985761140) | **+** | **+** |  | **+** |
| glycyl-glycine endopeptidase ALE-1 precursor (gi985761141) | **+** | **+** |  | **+** |
| hypothetical protein (gi985761142) | **+** | **+** |  | **+** |
| hypothetical protein (gi985761143) | **+** | **+** |  | **+** |
| hypothetical protein (gi985761144) | **+** | **+** |  | **+** |
| hypothetical protein (gi985761145) | **+** | **+** |  | **+** |
| hypothetical protein (gi985761146) | **+** | **+** |  | **+** |
| hypothetical protein (gi985761147) | **+** | **+** |  | **+** |
| hypothetical protein (gi985761148) | **+** | **+** |  | **+** |
| lysis protein (gi985761149) | **+** | **+** |  | **+** |
| GDSL-like lipase/acylhydrolase (gi985761150) | **+** | **+** |  | **+** |
| hypothetical protein (gi119967858) | **+** | **+** |  | **+** |
| N-acetylmuramoyl-L-alanine amidase (gi985761154) | **+** | **+** |  | **+** |
| **attR ATTATTATAATT** | **+** | **+** |  | **+** |
