# Supplementary material for: Genomic Comparison of Highly Virulent, Moderately Virulent, and Avirulent Strains From a Genetically Closely-Related MRSA ST239 Sub-lineage Provides Insights Into Pathogenesis
Source: Front Microbiol. 2018 Jul 10;9:1531. doi: 10.3389/fmicb.2018.01531 (PMC6048232; doi:10.3389/fmicb.2018.01531)
Supplement: Supplementary file 4 [file Table_4.DOCX]

**Suppl.Table 4.** φSPβ-like protein comparison based on PHASTER annotation.

| **Gene Product** | **TW20** | **CMRSA6** | **CMRSA3** | **M92** |
| --- | --- | --- | --- | --- |
| ORF073 (gi66396175) | + | + |  | + |
| hypothetical protein (gi162290174) | + | + |  | + |
| hypothetical protein (gi526244934) | + | + |  | + |
| hypothetical protein | + | + |  | + |
| minor head protein (gi431809708) | + | + |  | + |
| transposase mutator family (gi985761243) | + | + |  | + |
| hypothetical protein | + | + |  | + |
| hypothetical protein | + | + |  | + |
| hypothetical protein | + | + |  | + |
| hypothetical protein (gi100005) | + | + |  | + |
| SPP1 family phage head morphogenesis protein (gi526118333) | + | + |  | + |
| hypothetical protein | + | + |  | + |
| putative head morphogenesis protein (gi971819966) | + | + |  | + |
