# Supplementary material for: Genomic Comparison of Highly Virulent, Moderately Virulent, and Avirulent Strains From a Genetically Closely-Related MRSA ST239 Sub-lineage Provides Insights Into Pathogenesis
Source: Front Microbiol. 2018 Jul 10;9:1531. doi: 10.3389/fmicb.2018.01531 (PMC6048232; doi:10.3389/fmicb.2018.01531)
Supplement: Supplementary file 5 [file Table_5.DOCX]

**Suppl.Table 5.** SaPI1 component comparison based on PHASTER annotation.

| **Gene Product** | **TW20** | **CMRSA6** | **CMRSA3** | **M92** |
| --- | --- | --- | --- | --- |
| **attL TTGAAAATAAAA** | + | + | + | + |
| ORF002 (gi66395166) | + | + | + | + |
| enterotoxin P (gi30043936) | + | + | + | + |
| enterotoxin type A precursor (gi118725111) | + | + | + | + |
| ORF006 (gi66395170) | + | + | + | + |
| ORF020 (gi66395184) | + | + | + | + |
| ORF019 (gi66395183) | + | + | + | + |
| hypothetical protein | + | + | + | + |
| ORF021 (gi66395185) | + | + | + | + |
| hypothetical protein (gi66395180) | + | + | + | + |
| ORF003 (gi66395167) | + | + | + | + |
| hypothetical protein (gi156603988) | + | + | + | + |
| ORF013 (gi66395177) | + | + | + | + |
| hypothetical protein (gi431809698) | + | + | + | + |
| ORF008 (gi66395172) | + | + | + | + |
| ORF015 (gi66395179) | + | + | + | + |
| hypothetical protein | + | + | + | + |
| hypothetical protein | + | + | + | + |
| ORF010 (gi66395174) | + | + | + | + |
| ORF014 (gi66395178) | + | + | + | + |
| Phage terminase (gi66395173) | + | + | + | + |
| **attR TTGAAAATAAAA** | + | + | + | + |
